# Role of vanadium ions, oxygen vacancies, and interstitial zinc in room temperature ferromagnetism on ZnO-V_2_O_5_ nanoparticles

**DOI:** 10.1186/1556-276X-9-169

**Published:** 2014-04-07

**Authors:** Sion F Olive-Méndez, Carlos R Santillán-Rodríguez, Ricardo A González-Valenzuela, Francisco Espinosa-Magaña, José A Matutes-Aquino

**Affiliations:** 1 Centro de Investigación en Materiales Avanzados, S. C., CIMAV, Av. Miguel de Cervantes 120, Complejo Industrial Chihuahua, Chihuahua, Chihuahua 31109, Mexico; 2 Honeywell Manufacturas de Chihuahua, S de RL de CV, Chihuahua Chihuahua, 31136, Mexico

**Keywords:** Vanadium ions, Oxygen vacancies, Interstitial zinc, Room temperature ferromagnetism, V-doped ZnO

## Abstract

In this work, we present the role of vanadium ions (V^+5^ and V^+3^), oxygen vacancies (V_O_), and interstitial zinc (Zn_i_) to the contribution of specific magnetization for a mixture of ZnO-V_2_O_5_ nanoparticles (NPs). Samples were obtained by mechanical milling of dry powders and ethanol-assisted milling for 1 h with a fixed atomic ratio V/Zn?=?5% at. For comparison, pure ZnO samples were also prepared. All samples exhibit a room temperature magnetization ranging from 1.18?×?10^−3^ to 3.5?×?10^−3^ emu/gr. Pure ZnO powders (1.34?×?10^−3^ emu/gr) milled with ethanol exhibit slight increase in magnetization attributed to formation of Zn_i_, while dry milled ZnO powders exhibit a decrease of magnetization due to a reduction of V_O_ concentration. For the ZnO-V_2_O_5_ system, dry milled and thermally treated samples under reducing atmosphere exhibit a large paramagnetic component associated to the formation of V_2_O_3_ and secondary phases containing V^+3^ ions; at the same time, an increase of V_O_ is observed with an abrupt fall of magnetization to *σ*?~?0.7?×?10^−3^ emu/gr due to segregation of V oxides and formation of secondary phases. As mechanical milling is an aggressive synthesis method, high disorder is induced at the surface of the ZnO NPs, including V_O_ and Zn_i_ depending on the chemical environment. Thermal treatment restores partially structural order at the surface of the NPs, thus reducing the amount of Zn_i_ at the same time that V_2_O_5_ NPs segregate reducing the direct contact with the surface of ZnO NPs. Additional samples were milled for longer time up to 24 h to study the effect of milling on the magnetization; 1-h milled samples have the highest magnetizations. Structural characterization was carried out using X-ray diffraction and transmission electron microscopy. Identification of V_O_ and Zn_i_ was carried out with Raman spectra, and energy-dispersive X-ray spectroscopy was used to verify that V did not diffuse into ZnO NPs as well to quantify O/Zn ratios.

## Background

The complex mechanisms that allow ferromagnetic order at room temperature in diluted magnetic oxides (DMO) are a controversial subject as magnetic behavior is strongly dependent on the synthesis method and it is very difficult to obtain reproducible homogeneity on samples. It has been widely supported that ferromagnetism is originated by structural defects [1,2], mainly oxygen vacancies [3], but there exist some other structural defects such as interstitial cations [1,4], cation vacancies [5], impurities [6], and if we consider so, the common doping with 3d ions [7]. It has been shown theoretically and experimentally ([8] and references there in, [9]) that almost all of these defects have magnetic moment. On the other hand, some other systems report the absence of room temperature ferromagnetism on the same material combination. Coey et al. reported the construction of a phase diagram [10] for DMO, including percolation thresholds for oxygen vacancies (V_O_) and doping cations. Depending on the combination of these important defects, ferromagnetic, paramagnetic, or antiferromagnetic order can be presented on semiconducting or insulating oxides. Structural disorder can also be present in epitaxial thin films where crystalline order does not mean absence of Schottky and Frenkel defects. Epitaxial films are normally grown under thermodynamic equilibrium, avoiding an excessive formation of punctual defects higher than that intrinsically found: interstitial cations or V_O_ in ZnO, TiO_2_, or SnO_2_.

The most popular mechanism for ferromagnetic order in DMO is the bound magnetic polaron (BMP) where a trapped electron at the site of the V_O_, with a hydrogenic radius (0.4 to 0.6 nm), intercepts and polarizes the magnetic moment from 3d ions creating ferromagnetic order. Percolation of such BMPs creates a spin-polarized impurity band. The polarization of this band depends on the energetic overlapping with the spin split 3d bands of the cation. This is a reason which holds that no ferromagnetism would be expected for certain systems such as SnO_2_: Sc, Ti, and Zn [3] or ZnO: Cr [11]. On the other hand, ferromagnetism evidence on SnO_2_:Zn nanorods [12] was recently reported. It was proposed that substitutional Zn induced the formation of Sn_i_ defects to which is attributed the magnetic moment. This model is reinforced by theoretical calculations carried out by several groups [13,14]. The model used to refer the origin of magnetism based on interstitial cations is named BMP’ [15]. Structural defects do not mean partially amorphous material, but a concentration of punctual defects in monocrystalline structures.

In this paper, we report results concerning the structural and magnetic behavior of pure ZnO NPs milled under different conditions, and on the second part, we present a complete analysis of ZnO-V_2_O_5_ NPs, getting a clear conclusion about the role of each structural defect.

## Methods

Samples were obtained by mechanical milling using a high-energy SPEX mill (Spex Industries, Inc., Metuchen, NJ, USA) for 1, 8, and 24 h on a polymer jar with yttrium-stabilized zirconia balls. Powders 99.9% ZnO and 99.6% V_2_O_5_ (both from Sigma-Aldrich, St. Louis, MO, USA) were used on the stoichiometric proportion to have 5% at. of V atoms against the total amount of metallic atoms. Also, pure ZnO powders were milled for 1 h with and without ethanol to evaluate the contribution from interstitial zinc (Zn_i_) to the magnetic moment of the samples. Thermal treatment under reducing atmosphere (TT), a mixture of Ar:H_2_ [10:1], at 680°C for 1 h was applied to some of the obtained samples, a temperature barely higher than 672°C, which is the V_2_O_5_ melting point. This temperature was selected to ensure reaction between H_2_ and O from ZnO to produce V_O_. Magnetic *σ*(H) measurements were performed for all samples with a physical properties measuring system (PPMS) from Quantum Design (San Diego, CA, USA) at room temperature and an applied field of 2 T. Structural characterization was obtained from X-ray diffraction patterns (XRD). Chemical composition was identified by energy-dispersive X-ray spectroscopy (EDS) from EDAX in a transmission electron microscope (TEM) and in form of green compressed pellets in a scanning electron microscope (SEM). Micro-Raman spectroscopy was used to identify the presence of V_O_ and Zn_i_.

To name the samples, we use the following nomenclature: for ZnO-V_2_O_5_ samples, a number followed by letter *h* will be used to identify milling time. Ethanol-milled samples will have the suffix .*Et*, while dry milled samples do not have any suffix. Thermally treated samples will have. *Cal* suffix. Sample ZnO.Com represents commercial ZnO powder without any treatment. For example, sample 1 h.Et.Cal is a mixture of ZnO and V_2_O_5_ milled for 1 h with ethanol followed by TT, while ZnO.Et is pure ZnO ethanol-milled for 1 h and ZnO is 1-h dry milled ZnO.

## Results and discussion

### Pure ZnO nanoparticles

**Figure 1:**
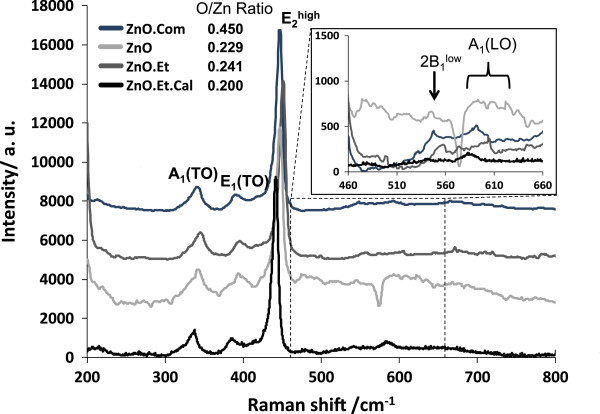
**Raman spectra of pure ZnO samples under different synthesis conditions.** Samples ZnO.Com, ZnO.Et, ZnO, and ZnO.Et.Cal. Sample ZnO (dry milled) has very different behavior than the rest of the samples; additional peaks are attributed to Zn_i_ impurity complexes.

Pure ZnO NPs were mechanically milled for 1 h with and without ethanol, samples ZnO.Et and ZnO, respectively. XRD patterns (not shown) for these samples and also from sample ZnO.Com show the wurtzite crystal structure; the only difference is related to the peak width. Using Scherrer formula, NPs from sample ZnO have an average size of 26 nm, while samples ZnO.Et and ZnO.Et.Cal measure 42 nm. Particles from sample ZnO.Com have an average size of 5 μm. The effect of mechanical milling on the creation of structural defects such as Zn_i_ and V_O_ on the NPs was evaluated by micro-Raman spectroscopy, as shown in Figure 1 for all samples. The modes corresponding to bulk ZnO that appear in our NPs are E_2_(high), A_1_(TO), and E_1_(TO), which are representative of a wurtzite structure. Of particular interest are A_1_ modes that are related to defects such as V_O_ and Zn_i_. On sample ZnO, A_1_(LO) mode at 590 cm^−1^ has the higher intensity that can be attributed to Zn_i_ and not to V_O_ as the sample was dry milled, and oxygen atoms at the surface limit formation of these latest defects. Spectra from samples ZnO.Com and ZnO.Et are very similar; only a reduction on the intensity of the peaks and a small shift are observed, assuming that only a change on the surface bonds of the NPs attributed to size change is reflected. Zn_i_ has a diffusion barrier of 0.57 eV [16] that makes it unstable at room temperature. However, it has been proposed that complexes involving N impurities could be stable at room temperature [17]. Ethanol milling avoided the adhesion of oxygen atoms at the surface of the NPs; thus, V_O_ concentration may remain stable. The effect of dry milling, ethanol milling, and TT on the stoichiometry of the samples is reflected on the O/Zn ratios obtained from EDS (Figure 1 next to sample labels).

**Figure 2:**
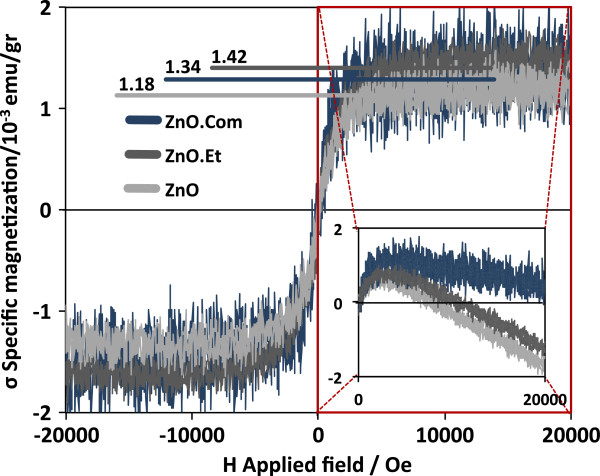
**Magnetic *σ*(H) loops performed at room temperature compared with commercial powders.** The increase of magnetization on sample ZnO.Et is attributed to formation of Zn_i_, while its reduction on sample ZnO is attributed to a reduction of V_O_. The inset represents the first and fourth quadrants including the diamagnetic component of M(H) loops as obtained from the PPMS. The lower value of the diamagnetic component on sample ZnO.Com suggests that Zn_i_ is randomly distributed in the whole particle.

Magnetic *σ*(H) loops, for all samples except for ZnO.Et.Cal, are shown in Figure 2 after subtraction of all diamagnetic components arising from the container and from nonferromagnetic ZnO. Sample ZnO.Com is expected to be completely diamagnetic; however, it has a magnetization of 1.34?×?10^−3^ emu/gr, attributed to a small amount of Zn_i_ and impurities of the material, as it is not a high-purity material. The inset of Figure 2 shows the first and fourth quadrant of the as-measured *σ*(H) loops; the lower absolute value of the slope of the diamagnetic component for sample ZnO.Com can be interpreted as concentration of randomly distributed impurities and Zn_i_ leading to a small diamagnetic component of ZnO. The increase of the absolute value of the slope after milling implies atom diffusion that increases the pure diamagnetic ZnO in the core of the NPs and a significant increase of Zn_i_ defects at the shell that are the sources of magnetic moment. For sample ZnO, oxygen from air during milling is in direct contact with NP surface; this implies a chemical potential of O_2_ that reduces the concentration of V_O_. Even if milling induces structural disorder and thus increase of Zn_i_, the total amount of V_O_, which mediates ferromagnetic order, decreases and then magnetization falls to 1.18?×?10^−3^ emu/gr.

For sample ZnO.Et, the O_2_ chemical potential is eliminated as the NPs are surrounded by ethanol molecules. Then, the amount of V_O_ is kept constant while milling increases the concentration of Zn_i_ (source of magnetic moment); as a consequence, magnetization increases from 1.34?×?10^−3^ (ZnO.Com) to 1.42?×?10^−3^ emu/gr. There exist some reports that attribute ferromagnetic signal in DMO only to V_O_, but with these defects even if they have magnetic moment (as a consequence of antiferromagnetic coupling with the sources of magnetism: interstitial cations of 3d dopants [18,19]), the role of V_O_ is only to mediate ferromagnetic order between magnetic moment sources and not to produce magnetic signal. For pure oxide systems, the used model is the BMP’. Our samples were used to confirm the existence of Zn_i_ defects at which we attribute the ferromagnetic enhancement magnetization by ethanol-assisted mechanical milling.

### ZnO-V_2_O_5_ nanoparticles

**Figure 3:**
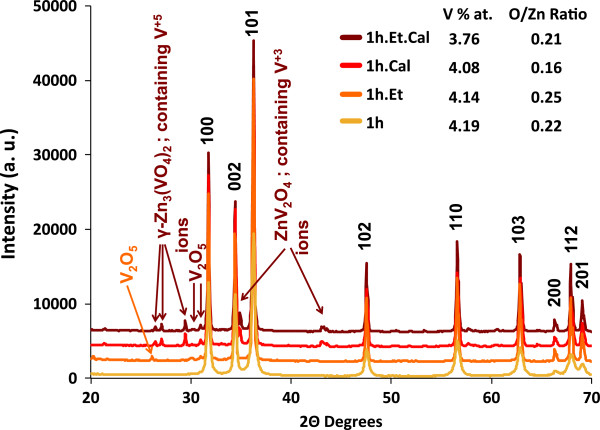
**XRD patterns for all ZnO-V**_**2**_**O**_**5 **_**samples showing the wurtzite structure of ZnO.** Additional peaks corresponding to V_2_O_5_, and secondary phases for some milling and TT processes. Near the sample labels, qualitative stoichiometric features of the samples are presented.

Identification of ZnO, V_2_O_5_, and secondary phases of all ZnO-V_2_O_5_ samples was carried out by XRD patterns shown in Figure 3. One of the most stable V oxides besides V_2_O_3_ is V_2_O_5_; both of them have affinity to form secondary phases with ZnO [20]. On sample 1 h, only the wurtzite structure of ZnO is observed, suggesting that dry milling reduces the size of V_2_O_5_ powders in order to make them undetectable for XRD. Using Scherer formula, ZnO NPs on this sample have an average size of 24 nm, while NPs from sample 1 h.Et (and samples after TT) have an average size of 45 nm, demonstrating that ethanol-assisted milling is more gentle with powders; also, small peaks corresponding to V_2_O_5_ are found on XRD pattern of sample 1 h.Et. Diffraction patterns of samples after TT (1 h.Cal and 1 h.Et.Cal) reveal the existence of V_2_O_5_ and the formation of γ-Zn_3_(VO_4_)_2_ and ZnV_2_O_4_ secondary phases which are the products of the reaction of ZnO with V_2_O_5_ and V_2_O_3_ after TT [20]. On the same figure, next to each sample label, the chemical composition features obtained by EDS - the V at. % and the O/Zn ratio - are shown; the last one reduces after each TT, demonstrating an increase of V_O_ concentration.

**Figure 4:**
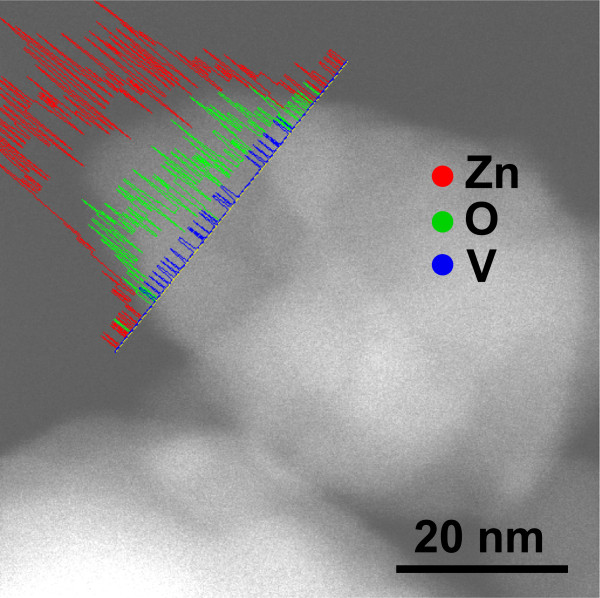
**TEM micrograph of a NP from sample 1 h.** The continuous V profile demonstrates that V atoms are surrounding the ZnO NP and no V diffusion into the NP is observed.

Figure 4 is a TEM micrograph of a NP from sample 1 h where the nominal V composition is 5% at. EDS line profiles of Zn, O, and V were obtained along the NP where the V profile is a constant line without any intensity change even in the thicker zones of the NP; we suggest that V oxide NPs are surrounding the ZnO NP. Milling process only reduces the particle size and favors the contact between both oxides at the surface of ZnO NPs, but no V diffusion is observed into the ZnO.

**Figure 5:**
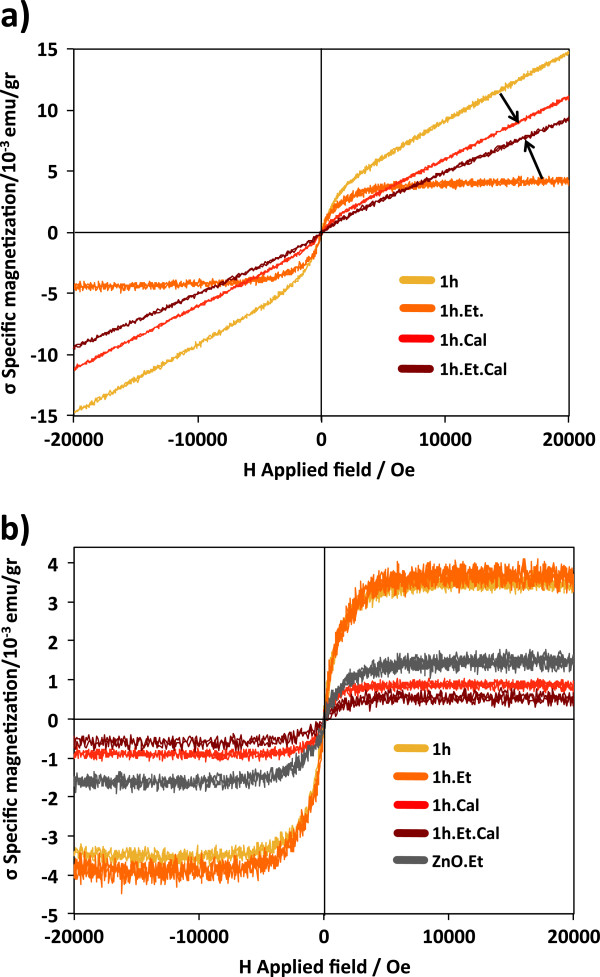
**Magnetization loops performed at room temperature showing paramagnetic and ferromagnetic components. (a)** Specific magnetization loops *σ*(H) for all ZnO-V_2_O_5_ samples after subtracting the diamagnetic component from the container. A strong paramagnetic component appears on samples 1 h, 1 h.Cal, and 1 h.Et.Cal which is attributed to the formation of V_2_O_3_ on sample 1 h, and secondary phases containing V^+3^ ions on samples 1 h.Cal and 1 h.Et.Cal. The arrows show how the paramagnetic component changes after TT. **(b)** Ferromagnetic components produced by V^+5, +3^ ions and V_O_ near the surface of the ZnO NPs to form BMPs.

Magnetic *σ*(H) loops for all 1-h milled samples are shown in Figure 5a. Sample 1 h has a very strong paramagnetic component that only can be attributed to V_2_O_3_ formation, which has a paramagnetic susceptibility equal to 13.184?×?10^−6^ cm^3^/gr which is larger than that of V_2_O_5_, *Χ*_V2O5_?=?0.703?×?10^−6^ cm^3^/gr. It is possible that V ions were reduced through the reaction V^+5^?+?2*e*^−^?→?V^+3^ where the electrons can be taken from the free electron pairs of oxygen from air, representing a chemical potential for this reaction. The spin-only magnetic moment of V^+3^ is 2.83 μ_B_/ion, while V^+5^ should be completely diamagnetic. Sample 1 h.Et has a weak paramagnetic component attributed to the lack of reduction of V^+5^ ions (absence of oxygen surrounding V_2_O_5_ NPs); by XRD, we only detect V_2_O_5_. These paramagnetic-diamagnetic components for samples 1 h and 1 h.Et are consistent with the previous explanation from XRD patterns, where almost all V_2_O_5_ is transformed in very small V_2_O_3_ NPs for sample 1 h with high paramagnetic susceptibility, while sample 1 h.Et (less aggressive milling) has a significant amount of V_2_O_5_, reducing the value of the paramagnetic slope in Figure 5b.

Samples with TT have a reduction of the O/Zn ratio as a consequence of the creation of V_O_; these ratios are semiqualitative as EDS is not a completely quantitative technique. There is also a reduction of the V concentration as a consequence of V_2_O_5_ evaporation.

Secondary phase formation containing V^+3^ ions for samples with TT is also supported by the high positive susceptibility measured on samples; the arrows in Figure 5a indicate the direction in which the susceptibility from samples 1 h and 1 h.Et has changed after TT, supporting the idea that γ-Zn_3_(VO_4_)_2_ and ZnV_2_O_4_ are formed during TT and/or cooling and not during milling. A combination of diamagnetic susceptibility from ZnO and paramagnetic susceptibility from γ-Zn_3_(VO_4_)_2_ and ZnV_2_O_4_ contributes to the approached value (arrows in Figure 5a). The paramagnetic change is stronger on sample 1 h.Et.Cal, which has lower V_2_O_3_ content after milling; then, the TT reduced some of the V^+5^ to V^+3^ ions and both of the secondary phases are formed. For sample 1 h, the behavior turns on the opposite way; susceptibility has a slight decrement suggesting separation of V_2_O_3_ NPs from ZnO surface to form secondary phases and V_2_O_5_.

Ferromagnetic components from typical DMO mechanisms for all samples are shown in Figure 5b. Samples 1 h and 1 h.Et have the highest specific magnetizations *σ*?~?3.5?×?10^−3^ emu/gr, but as sample 1 h has the largest paramagnetic component, attributed to V_2_O_3_, we can assume that not all V^+5^ or V^+3^ contribute to the ferromagnetic moment on the samples. Usually high doping concentration of magnetic ions forms antiferromagnetic complexes [21]; this is the reason that lower ion concentration produces the highest magnetic moment per doping ion. As V has a very low solubility limit on ZnO?~?0.2% [22], secondary phases are more easily formed instead of promotion of V diffusion into the ZnO matrix. After TT magnetization decays to *σ*?~?0.7?×?10^−3^ emu/gr, which has been already explained with the formation of secondary phases and is also due to a reduction of structural defects on ZnO, NPs from sample 1 h increase their average size by coalescence to reduce their surface free energy and a reorganization of the surface is promoted by atom diffusion, reducing the sources of magnetism; at the same time, reaction between ZnO and V oxides produces secondary phases, reducing the number of ZnO/V interfaces.

**Figure 6:**
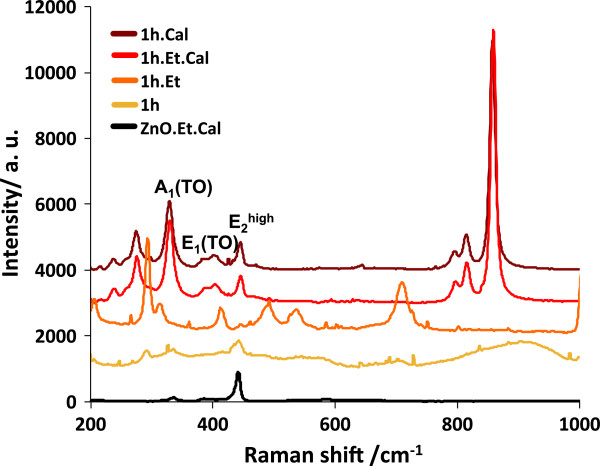
**Raman spectra of ZnO-V**_**2**_**O**_**5 **_**nanoparticles with and without thermal treatment.** Samples 1 h.Cal and 1 h.Et.Cal exhibit a strong paramagnetic component attributed to the formation of secondary phases containing V^+3^ ions. The peaks in the intervals 200 to 360 and 780 to 1,000 cm^−1^ are attributed to ZnV_2_O_4_ phase. The weak and broad peaks for sample 1 h centered at 420 and 900 cm^−1^ are attributed to amorphous material linked to V^+3^ ions.

Raman spectra of ZnO-V_2_O_5_ NPs are shown in Figure 6; for samples 1 h.Et and 1 h.Et.Cal, it is very clear that the set of peaks at 200 to 380 and 780 to 1,000 cm^−1^ belong to ZnV_2_O_4_ phase, and this result is consistent with previous XRD patterns of the same samples. Sample 1 h has two peaks (weak and broad) located near the regions were the ZnV_2_O_4_ phase was identified. Peaks from sample 1 h.Et does not match with any of the previous cases, as this sample is the only one that does not exhibit paramagnetic component; peaks must correspond to V_2_O_5_ NPs. All spectra are compared with a pure ZnO sample milled with ethanol and thermally treated.

**Figure 7:**
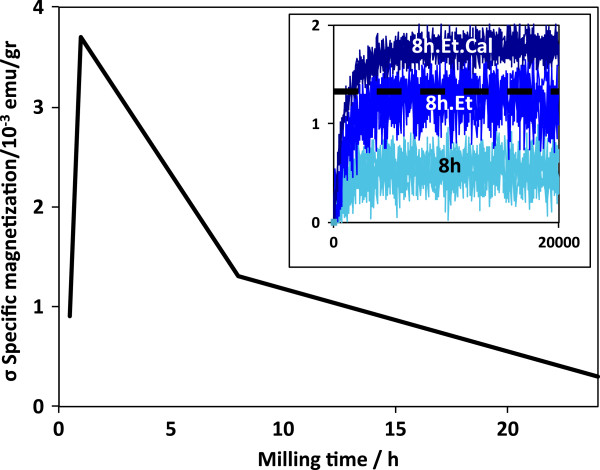
**Variation of saturation magnetization depending on milling time.** The maximum for our synthesis parameters was found around 1 h. The inset shows how the TT increased the magnetic moment for samples milled for 8 h. Probably, long-time milling contributes to reduce intrinsic V_O_ on ZnO, thus reducing the defects that mediate ferromagnetic order.

Dry milling produces a size reduction of V_2_O_5_ powders, but no phase change is involved. On sample 1 h, a small amount of V_2_O_5_ is used to produce magnetic moment; the rest is transformed to V_2_O_3_. We conclude that there exists a threshold concentration for which larger concentrations of magnetic ions do not help to increase the magnetic signal. No antiferromagnetic coupling is observed. To verify this fact, powders where milled with and without ethanol for 8 h to reduce particle size and increase the ZnO/V interface area; then, TT was applied for the 8 h.Et sample. Average particle sizes determined by Scherer formula are 19 and 45 nm for samples 8 h and 8 h.Et, respectively, reducing the particle size only for dry milled sample. Magnetization *σ*(H) loops (first quadrant) are shown in the inset of Figure 7. Here the TT enhances the saturation magnetization, but all magnetizations are smaller than that of sample 1 h.Et. Probably, long-time milling contributes to reduce intrinsic V_O_ on ZnO by reduction of V^+5^ ions. In Figure 7, a variation of saturation magnetization depending on milling time is shown. The maximum depends on the mass of the milled powders, the amount of ethanol, the size of the jar, and the number and size of the milling media, reinforcing the idea that ferromagnetism in DMO is not trivial and synthesis conditions are critical in order to maximize magnetic moment of the samples.

## Conclusions

We prepared pure ZnO and a mixture of ZnO and V_2_O_5_ NPs by mechanical milling in different conditions: dry and ethanol-assisted milling. From Raman spectra of the pure ZnO dry milled sample, the increase of the signal of the A_1_(LO) mode, related to structural defects such as Zn_i_, supports the fact that this defect is the source of magnetic moment as the sample has higher magnetization than that of commercial ZnO. On the other hand, dry milled samples exhibit a reduction of magnetization; even if milling increases the concentration of Zn_i_, the exposure of the powders to oxygen from air during milling reduces the amount of V_O_, which mediates ferromagnetic order between Zn_i_. The coupling between Zn_i_ through V_O_ corresponds to the BMP’ model.

For the ZnO-V_2_O_5_ system, it was proven that V^+5^ ions added at the surface of the ZnO NPs form BMPs, increasing the magnetization from 1.42?×?10^−3^ to 3.5?×?10^−3^ emu/gr, demonstrating that V ions produces magnetic order in the system ZnO:V. TT induced the formation of ZnV_2_O_4_ secondary phase, containing V ^+3^ ions, which is paramagnetic. V^+3^ ions are also present on ZnO-V_2_O_5_ dry milled sample as shown by a weak and broad peak on Raman spectra on the interval 750 to 1,000 cm^−1^, supporting the idea that dry milling, in some form, reduces the charge of some ions from V^+5^ to V^+3^. After TT, the amount of V_O_ was increased but magnetization falls to 0.7?×?10^−3^, demonstrating that the intrinsic amount of V_O_ on ZnO is enough to mediate ferromagnetic order.

## Competing interests

The authors declare that they have no competing interests.

## Authors’ contributions

SFOM made all the NP samples, performed data interpretation, and worked on the manuscript. CRSR performed magnetic measurements. RAGV participated in Raman interpretation and financial facilities. FEM worked on the manuscript, and JAMA participated in acquiring the magnetic loops facilities. All authors read and approved the final manuscript.

## Authors’ information

All authors work at CIMAV Chihuahua, with the exception of RAGV who works at Honeywell Chihuahua as a design engineer. SFOM is working as a researcher in the field of nanostructured and magnetic materials. CRSR is working as a technician in charge of several magnetic measuring techniques. FEM is a professor working with theoretical simulation, and JAMA is a professor working with a wide variety of magnetic materials.
